# Correction: Loss of PINK1 Increases the Heart's Vulnerability to Ischemia-Reperfusion Injury

**DOI:** 10.1371/annotation/94fd6502-4b2d-409c-8836-66fe6ebc03ab

**Published:** 2013-06-26

**Authors:** Hilary K. Siddall, Derek M. Yellon, Sang-Bing Ong, Uma A. Mukherjee, Niall Burke, Andrew R. Hall, Plamena R. Angelova, Marthe H. R. Ludtmann, Emma Deas, Sean M. Davidson, Mihaela M. Mocanu, Derek J. Hausenloy

There were errors in the order of labels and legends. 

The labels and legends for Figures 5 and 6 were switched. The label and legend that appear under Figure 5 belong under Figure 6, and the label and legend that appear under Figure 6 belong under Figure 5. The figures themselves are in correct order. 

In addition, the labels and legends for Figures 7 and 8 were incorrectly switched. The label and legend that appear under Figure 7 belong under Figure 8, and the label and legend that appear under Figure 8 belong under Figure 7. The figures themselves are in correct order.

